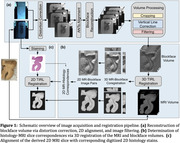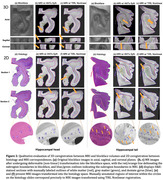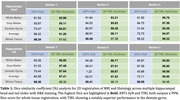# Achieving micron‐level precision MR‐Histology correlation for validating Alzheimer’s disease biomarkers in the human hippocampus

**DOI:** 10.1002/alz.092110

**Published:** 2025-01-09

**Authors:** Yixin Wang, William Hai Dang Ho, Istvan N. Huszar, Hossein Moein Taghavi, Jeffrey Nirschl, Samantha Leventis, Philip Schlömer, Markus Axer, Wei Shao, Mirabela Rusu, Phillip DiGiacomo, Marios Georgiadis, Michael Zeineh

**Affiliations:** ^1^ Stanford University, Stanford, CA USA; ^2^ Harvard Medical School, Boston, MA USA; ^3^ Forschungszentrum Jülich, Jülich, Jülich Germany; ^4^ University of Florida, Gainesville, FL USA

## Abstract

**Background:**

MRI offers potential noninvasive detection of Alzheimer's micropathology. The AD hippocampus exhibits microscopic pathological changes such as tau tangles, iron accumulation and late‐stage amyloid. Validating these changes from ultra‐high‐resolution ex‐vivo MRI through histology is challenging due to nonlinear 3D deformations between MRI and histological samples. We aim to address the challenge by a pipeline (Figure 1) for precise alignment of post‐mortem MRI data with histological images.

**Method:**

A human hippocampal specimen was dissected from a formalin‐fixed human brain and divided into hippocampal head and tail. We obtained high‐resolution multi‐echo gradient echo ex vivo MRI of each using a 7T Bruker scanner. The specimens were paraffin‐embedded and sectioned using a Leica Histocore Nanocut R microtome. An optical image of the tissue block's surface was captured before each section was cut (Blockface image) using a polarized filter at Brewster’s angle. The obtained 2D blockface images were aligned and filtered into 3D blockface volumes. These were registered with MRI using either Tensor Image Registration Library (TIRL) with Modality Independent Neighbourhood Descriptor, or ANTs SyN registration. Subsequently, the 2D MRI slices corresponding to the stained histology slides were identified in the 3D volume and underwent 2D deformable registration with TIRL or ANTs. To quantify the registration accuracy, we segmented hippocampal volumes into white matter, grey matter, and dentate gyrus for MRI, blockface, and histology data separately.

**Result:**

When comparing 3D MR and blockface 3D image segmentations (Figure 2, top), the average Dice similarity coefficient of hippocampal head and tail were 86.2% and 86.76% with TIRL, and 83.91% and 78.26% with ANTs SyN. Evaluating 2D MR registration to histological images, TIRL achieves the average Dice coefficient of 94.12% and 96.95%. ANTs performs marginally better across the whole tissue mask, but TIRL shows superior performance in the internal features (Table 1). High‐fidelity alignment shows very small features such as focal hypointensities (Figure 2, bottom, blue circles) and the dentate granule cell layer (orange arrows).

**Conclusion:**

Our advanced correlative MRI‐histology pipeline achieves micron‐level precision coregistration methods at micron‐scale precision, propelling the development of MRI‐based AD biomarkers.